# The Successful Use of Neo Adjuvant Brentuximab Vedotin in the Treatment of BIA-ALCL

**DOI:** 10.1097/HS9.0000000000000501

**Published:** 2020-11-24

**Authors:** Rebecca L. Allchin, Katherine Wickenden, Simon Pilgrim, Issac Wilson-Morkeh, Fiona M. Miall

**Affiliations:** 1 Department of Hematology, University Hospitals, Leicester, United Kingdom; 2 Department of Breast Surgery, University Hospitals, Leicester, United Kingdom; 3 Department of Hematology, Kettering General Hospital, Kettering, United Kingdom.

Breast-implant associated anaplastic large cell lymphoma (BIA-ALCL) is an increasingly recognized diagnosis and is described in the WHO classification (2017).^1^ We present a case of disease with local infiltration managed with neo-adjuvant brentuximab vedotin (BV) monotherapy leading to a complete radiological response enabling successful complete excision with considerably less extensive surgery than initially anticipated. The patient remains in remission 18 months post-operatively.

**Figure 1:**
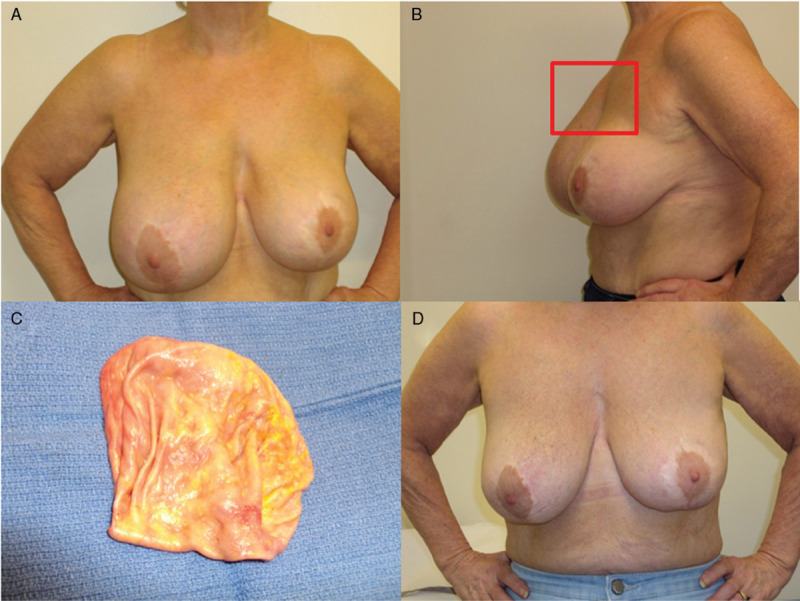
Pre-operative clinical photographs (A & B) with the swollen right breast due to the mass visible in the right upper quadrant (highlighted in B) and seroma. Capsule removed intact with thickening but no residual mass (C). Clinical photograph taken 19 days post-operatively (D).

A 65-year-old lady presented in early 2018 with swelling and a mass in the upper inner quadrant of the right breast (Fig. 1A and B). This was 9 years after bilateral subglandular breast augmentation with mastopexy with textured silicone implants She had no B symptoms. Examination showed periareolar and vertical lower pole scars on both breasts consistent with her previous surgery. The right breast was very swollen with a fixed mass at the second—third sternocostal junction. The mass felt fixed to the underlying chest wall.

She had a history of asthma and previous hysterectomy and two Caesarean sections. Regular medication included Salbutamol and Formoterol inhalers and Lymecycline. She was a long-term smoker but with the intention to stop (40 pack years) and drank alcohol rarely. She had a family history of cervical cancer in two sisters but no family history of lymphoproliferative disease.

**Figure 2:**
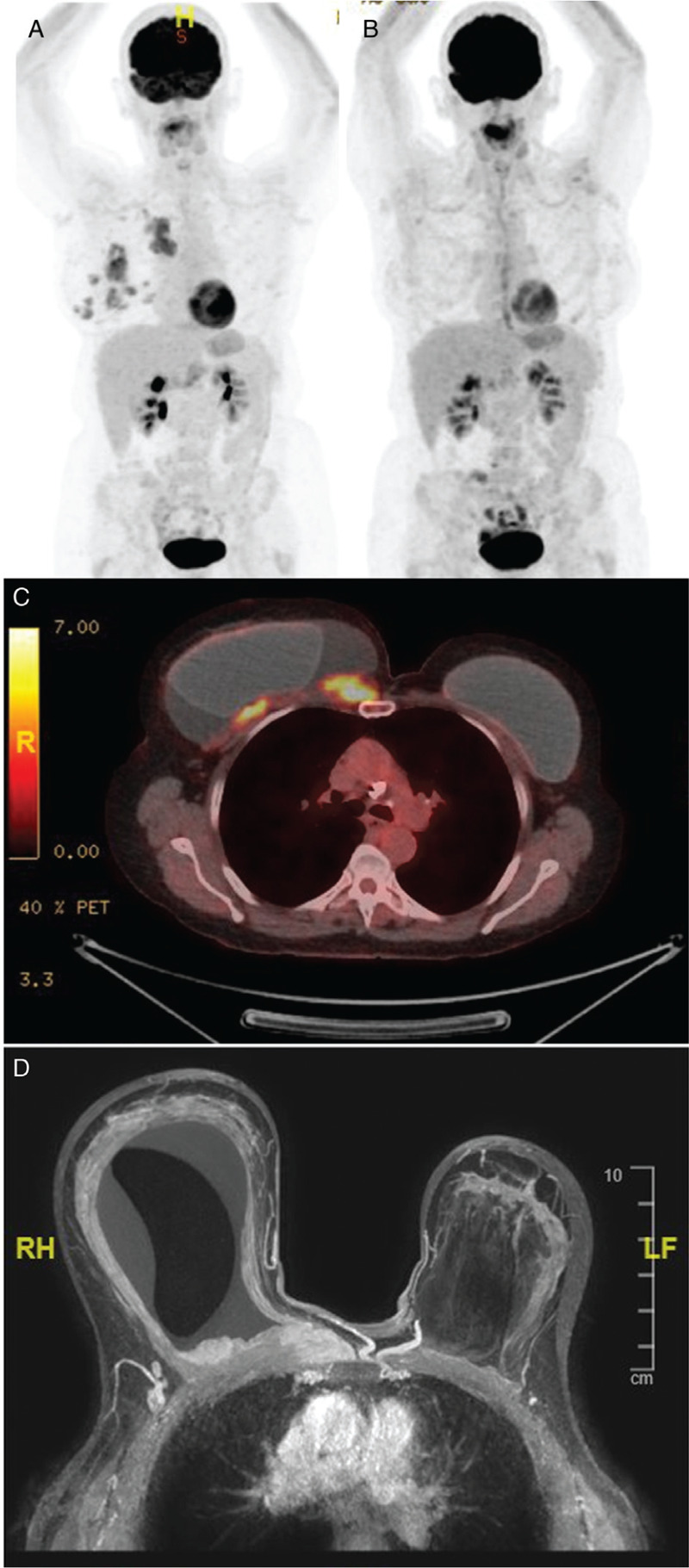
**Radiological images.** PET MIP images pre-treatment (A) and following 8 cycles of BV (B) demonstrating a complete metabolic response (Deauville 1). Pre-treatment axial image from PET-CT (C) showing the FDG-avid soft tissue mass and dynamic MIP MRI (D) demonstrating infiltration of the underlying muscle.

Initial aspirations of right breast seroma were non-diagnostic but suspicious for the presence of abnormal CD30+ cells. She went on to have CT and MRI imaging which showed significant fluid along with a 5 × 4 × 3 cm mass in the right upper quadrant with suggestion of infiltration of the underlying pectoral muscle (Fig. 2D). A subsequent core biopsy from the chest wall mass confirmed a diagnosis of BIA-ALCL showing an infiltrate of large atypical cells with irregular convoluted hyper-lobated nuclei and prominent nucleoli. They showed strong expression of CD30 and some staining with CD45 and EMA. The atypical cells were entirely negative with CD20, CD79a, CD5 and epithelial markers. Additional immunohistochemistry demonstrated positive staining with CD4 but no significant staining with ALK1, PAX5, CD2, CD7 or CD8. A clonal TCR gamma gene rearrangement was demonstrated by polymerase chain reaction.

At the time of diagnosis she had a normal full blood count and a lactate dehydrogenase of 172 iu/L and normal renal and liver function tests.

A PET-CT showed localized disease with infiltration beyond the capsule. The mass was FDG-avid with an SUV max of 7.2 with a liver reference of 3.2 and mediastinal reference of 2.6 (Fig. 2A and C). Bone marrow aspirate and trephine showed no evidence of involvement. This confirmed stage IIA (T4N0M0) disease due to the infiltration of lymphoma beyond the capsule. Unlike many lymphoproliferative diseases BIA-ALCL is staged using a TNM staging system as proposed by the MD Anderson in 2016, rather than the Ann Arbor staging system.

Surgical resection was considered as this is the standard of care for BIA-ALCL.^2^ On the basis of clinical and imaging findings it was felt that resection would require removal of the capsule with mastectomy and removal of underlying pectoralis major and possible removal of underlying ribs & sternum. This represented a significantly invasive procedure with a high risk of morbidity and requiring breast, thoracic and plastic surgeons to be present.

Given the extent of the surgery required to attempt a complete excision other options were explored.

Radiotherapy is not standard therapy in the neo-adjuvant setting and may have increased the complication rate of subsequent surgery. Neo-adjuvant chemotherapy was considered, reports of progression through CHOP and radiotherapy in this setting exist.^3^ After seeking national and international expert advice, we received reports of the successful use of BV in invasive BIA-ALCL to allow less invasive surgery (personal communication, Dr M Clemens) and a decision was made to use BV in this case.

After 4 cycles of BV a PET-CT showed a Deauville 1 metabolic response but significant residual soft tissue mass and further BV cycles were given. Following 8 cycles of BV the complete metabolic response continued now with significant reduction in the size of the mass (Fig. 2 A and B).

She went onto have bilateral implant removal and capsulectomy on the right with biopsy of the chest wall in January 2019. At surgery there was a clear plane between the capsule and the pectoralis major so no muscle or rib resection was required and mastectomy was avoided (Fig. 1C and D). Immunohistochemistry of seroma and chest wall biopsy showed no evidence of residual disease.

Given the excellent radiological response and absence of residual disease found at resection and confirmed by histological examination no further therapy with BV or radiotherapy was given.

She has had a good post-operative recovery, although did experience a myocardial infarction requiring stent placement and anticoagulation 2 months post-operatively. At her last clinic review she had some residual grade 1 neuropathy. She remained in a complete metabolic remission based on a PET-CT scan 8 months post-operatively and is now 18 months post-surgery with ongoing clinical remission.

BIA-ALCL is an uncommon condition which following the insertion of a textured breast implant, occurring with an estimated frequency of 1 to 3 cases per million implants at an average of 10 years. The majority of patients present with unilateral breast swelling and have localized disease (effusion only) with 10% to 15% presenting with locally invasive disease and less than 10% presenting with nodal or distant metastases.^2^

BIA-ALCL is generally associated with an excellent prognosis. Those presenting with localized disease, fluid collections confined to the capsule, can usually be cured with surgical resection. Five-year overall survival (OS) in this group has been reported at 100%. Those with evidence of locally advanced disease have higher rates of relapse after initial therapy and a five year OS of 75%.^4^ Complete surgical excision is associated with improved survival compared to partial capsulectomy.^3^ As such, standard of care in BIA-ALCL is resection with complete removal of the implant and capsulectomy, followed by systemic treatment in those with stage IIB or higher disease.^2^

The optimal management of those with locally invasive disease where extensive thoracic surgery would be required for complete excision, as in this case, is very unclear due to its rarity.

Chemotherapy options in BIA-ALCL are extrapolated from the treatment of systemic (sALCL) and cutaneous ALCL (cALCL). Response rates to standard cytotoxic chemotherapy such as CHOP in sALCL are higher in ALK+ cases than ALK- cases^5^ with many centers opting to consider escalation of therapy and autologous stem cell transplantation in 1st remission for cases of ALK- sALCL.^6^

BIA-ALCL is characteristically ALK- and complete surgical excision is recognized as an essential part of therapy with complete excision associated with excellent long-term outcomes.^3^ For cases which are stage IIB-IV or stage I-IIA with incomplete excision the best choice of adjuvant therapy remains unclear due to the lack of prospective data. Various options are recommended in the 2019 NCCN guidelines.^2^

Standard cytotoxic chemotherapy was considered (cyclophosphamide, doxorubicin, vincristine and prednisolone +/− etoposide; CHOP or CHOEP), however, 2 case series describe similar cases of BIA-ALCL which were refractory to neo-adjuvant CHOP.^3,7^

BV is an anti-CD30 antibody-drug conjugate with established efficacy as a single agent in systemic ALCL in the relapsed setting. Use of BV for chemotherapy-refractory BIA-ALCL has been reported^8,9^ and the toxicity of single-agent BV reported in the treatment of lymphoma is less than that expected with conventional cytotoxic chemotherapy.^5,10,11^

The ECHELON-2 trial has shown a benefit of BV in combination with cyclophosphamide, doxorubicin and prednisolone (BV-CHP) in up-front treatment of CD30+ peripheral T cell lymphomas (including sALCL)^12^ however this was not published at the time of this patient's presentation and treatment and included no cases of BIA-ALCL. This approach would not currently be funded in the UK for BIA-ALCL and has only recently been approved for use by the National Institute for Health and Care Excellence for use in untreated sALCL (August 2020).^13^

The need for neo-adjuvant therapy is not discussed in the NCCN guidelines and a UK case series from 2017 included only a single case where neo-adjuvant therapy was considered necessary.^2,7^ In this case initial therapy with CHOP was unsuccessful with disease progression after 3 cycles. BV monotherapy for 6 cycles led to a response prior to surgery.^7^ Case reports describing the effectiveness of BV monotherapy for residual disease following implant removal exist^9,14^ suggesting good disease control.

Adjuvant therapy with chemotherapy and/or radiotherapy is recommended for those with stage IIB or higher disease or when excision was incomplete.^2^ Although this case had extensive local infiltration prior to neo-adjuvant therapy histology showed no evidence of residual disease at the time of surgery confirming complete excision.

Our case demonstrates that where locally invasive disease would require major surgical intervention neoadjuvant therapy should be considered. This case shows an excellent response to BV monotherapy enabling complete resection of the implant with no residual disease demonstrated histologically or radiologically. It highlights the importance of multi-disciplinary management with communication between the breast, thoracic and plastic surgeons with the hemato-oncologists and histopathologists. Our patient tolerated BV with some grade 1 neuropathy and continues in a clinical remission 18 months post-operatively.
